# Results of anxiety disorders in a medical professional

**DOI:** 10.1192/j.eurpsy.2024.878

**Published:** 2024-08-27

**Authors:** P. Sarantuya, B. Putev, T. Myatav

**Affiliations:** ^1^Medical department, Etugen university; ^2^Avicenna science center, Ulaanbaatar, Mongolia

## Abstract

**Introduction:**

Anxiety is the most prevalent disease in the world. Symptoms of anxiety disorders affect everyone. The symptoms are worse after a long period of time and more severe disease than normal. A study was conducted to support the assumption that it is an opportunity to improve healthcare services.

**Objectives:**

1. Anxiety disorders of doctors and medical specialists of Selenge Province General Hospital
2. Determine the relationship between certain factors and certain factors of anxiety disorders

**Methods:**

The GAD7, SRQ20, and PHQ9, sleep system detection questionnaire methods issued by WHO for doctors of primary health care institutions were analyzed by analytical research snapshot model from 03.15 to 04.05, 2023.03.22. /1/01 was obtained and the survey was conducted.

**Results:**

In the study, 23-65-year-olds received medical care, and the average life expectancy was 37.05 years. 30% (27) of the respondents did not have anxiety disorders, 36.67% (33) had mild anxiety disorders, 18.89% (17) had moderate anxiety disorders, and 14.44% (13) had severe health problems. 6.67% (6) of the respondents had no depression, 10% (9) had very mild depression and could cope on their own, 24.44% (22) had moderate depression and could cope, and 27.78% (25) with healthy depression. 24.44% (22) had major depressive disorder and 6.67% (6) had major depressive disorder. According to correlation analysis, GAD7 score with SRQ20 stress score r=0.76 and PHQ9 mood score with r=0. the inverse association was statistically significant at p=0.00. PHQ9 depression score had a strong effect on SRQ20 stress score r=0.74, p=0.00, and GAD7 score r =0.46, p=0.0000. r=-0.40, p=0.00 had a moderate inverse relationship with age, and r=-0.24, p=0.00 had a weak inverse relationship with age. In linear regression analysis, the GAD7 anxiety disorder score increased by 48.8% (p=0.00) when fixed at one, which was statistically significant. In logistic regression analysis, PHQ9 depression score increased by 35.08% (p=0.01) per entry. In composite logistic regression analysis, the PHQ9 depression score was statistically significant OR=4.07 (p=0.01) multiplied by one.

**Conclusions:**

Doctors and medical professionals include psychological health research, testing, treatment, psychological counseling, and health care. Anxiety disorders are related to stress, depression, satisfaction, and age, while depressive disorders are anxiety disorders.

**Disclosure of Interest:**

None Declared

